# Automatic detection and prediction of epileptic EEG signals based on nonlinear dynamics and deep learning: a review

**DOI:** 10.3389/fnins.2025.1630664

**Published:** 2025-08-18

**Authors:** Shixiao Tan, Zhen Tang, Qiang He, Ying Li, Yuliang Cai, Jiawei Zhang, Di Fan, Zhenkai Guo

**Affiliations:** ^1^School of Medical and Bioinformatics Engineering, Northeastern University, Shenyang, China; ^2^School of Computer Science and Engineering, Northeastern University, Shenyang, China; ^3^School of Mathematics and Statistics, Liaoning University, Shenyang, Liaoning, China; ^4^Department of Vascular and Thyroid Surgery, The First Hospital of China Medical University, Shenyang, China; ^5^School of Mathematics and Statistics Science, Ludong University, Yantai, China

**Keywords:** epileptic seizures detection, nonlinear dynamics, epilepsy prediction, chaos theory, fractal analysis

## Abstract

Epilepsy is a neurological disorder affecting ~50 million patients worldwide (30% refractory cases) with complex dynamical behavior governed by nonlinear differential equations. Seizures severely impact patients' quality of life and may lead to serious complications. As a primary diagnostic tool, electroencephalography (EEG) captures brain dynamics through non-stationary time series with measurable chaotic and fractal properties. However, EEG signals are highly nonlinear and non-smooth, and conventional linear analysis methods limited by Fourier spectral decomposition cannot capture the inherent phase space dispersion and multifractal geometries of epileptic signals. In recent years, nonlinear dynamics methods such as chaos theory, fractal analysis, and entropy computation have provided new perspectives for EEG signal analysis, while deep learning approaches like convolutional neural networks and long short-term memory networks further enhance the robustness of dynamical pattern recognition through end-to-end nonlinear feature extraction. These methods reveal dynamic patterns in signals, thereby substantially improving epilepsy detection and prediction accuracy. This survey reviews research progress in automatic detection and prediction of epileptic EEG signals based on nonlinear dynamics and deep learning, evaluating key techniques including Lyapunov exponents, fractal dimensions, and entropy metrics. Results highlight three paradigm shifts, including the demonstrated superiority of nonlinear features in capturing preictal transitions, the critical role of attention mechanisms in processing long-range dependencies, and the significant advantages achieved by integrating nonlinear attributes with deep learning architectures for cross-patient generalization and noise suppression. Furthermore, this survey identifies persistent challenges including clinical translation barriers, algorithm performance trade-offs, and feature extraction/selection limitations. It emphasizes the need to integrate algebraic topology and graph convolutional deep learning to address multiscale dynamics, and proposes a unified framework for regulatory-compliant clinical translation that bridges the gap between research innovations and real-world clinical deployment, while outlining future research priorities focused on multimodal data fusion and regulatory-compliant validation frameworks.

## 1 Introduction

Epilepsy is a widespread neurological disorder characterized by recurrent episodes of abnormal brain electrical activity (Shao et al., 2025). Approximately 50 million people worldwide are estimated to suffer from epilepsy, with more than 200,000 new cases annually, making it the third most common neurological disorder after Alzheimer's disease and stroke (Mormann et al., 2007; Mormann and Andrzejak, 2016). During seizures, the normal pattern of neuronal activity is disrupted, potentially resulting in motor dysfunction, bowel or bladder control disorders, and loss of consciousness (Kuang et al., 2024). These symptoms not only affect patients' daily lives but can also increase their risk of serious complications, including sustained state epilepticus (SCLES), fractures, and even sudden unexpected death in epilepsy (SUDEP) (Nasehi and Pourghassem, 2012; Chen et al., 2019). Therefore, achieving timely and accurate seizure prediction and diagnosis would help prevent these potentially serious consequences. Electroencephalography (EEG) serves as the primary tool for diagnosing and monitoring epilepsy (Solaija et al., 2018), capable of recording electrical signals from neuronal activity in the brain (Wang et al., 2017). However, epileptic EEG signals are highly nonlinear and non-smooth, with complex and variable seizure patterns (Andrzejak et al., 2012), which limits traditional signal processing methods such as Fourier transform and wavelet transform in feature extraction and pattern recognition.

**Figure 1:**
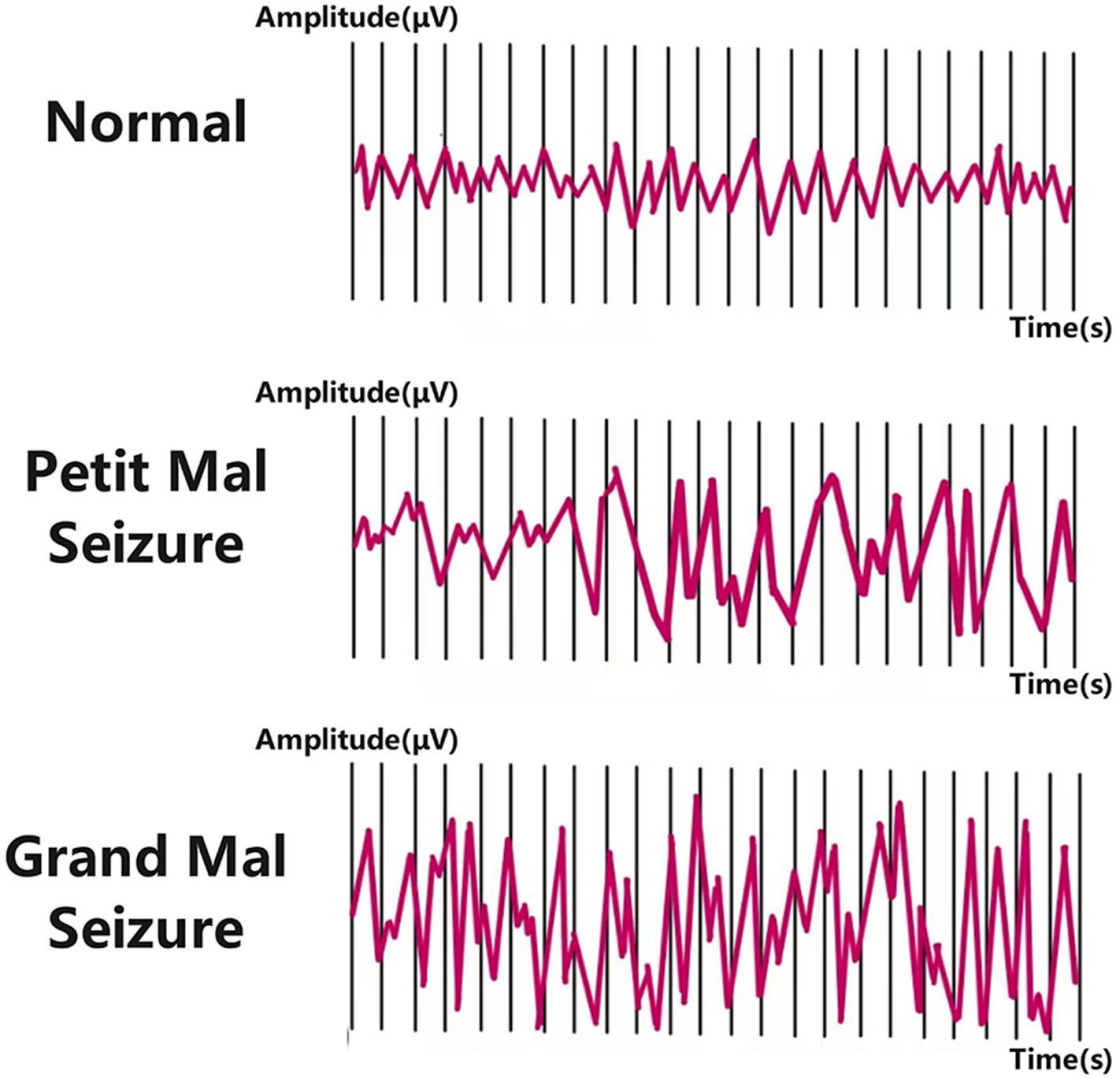
Epileptic seizures: EEG waveforms in different stages.

**Figure 2:**
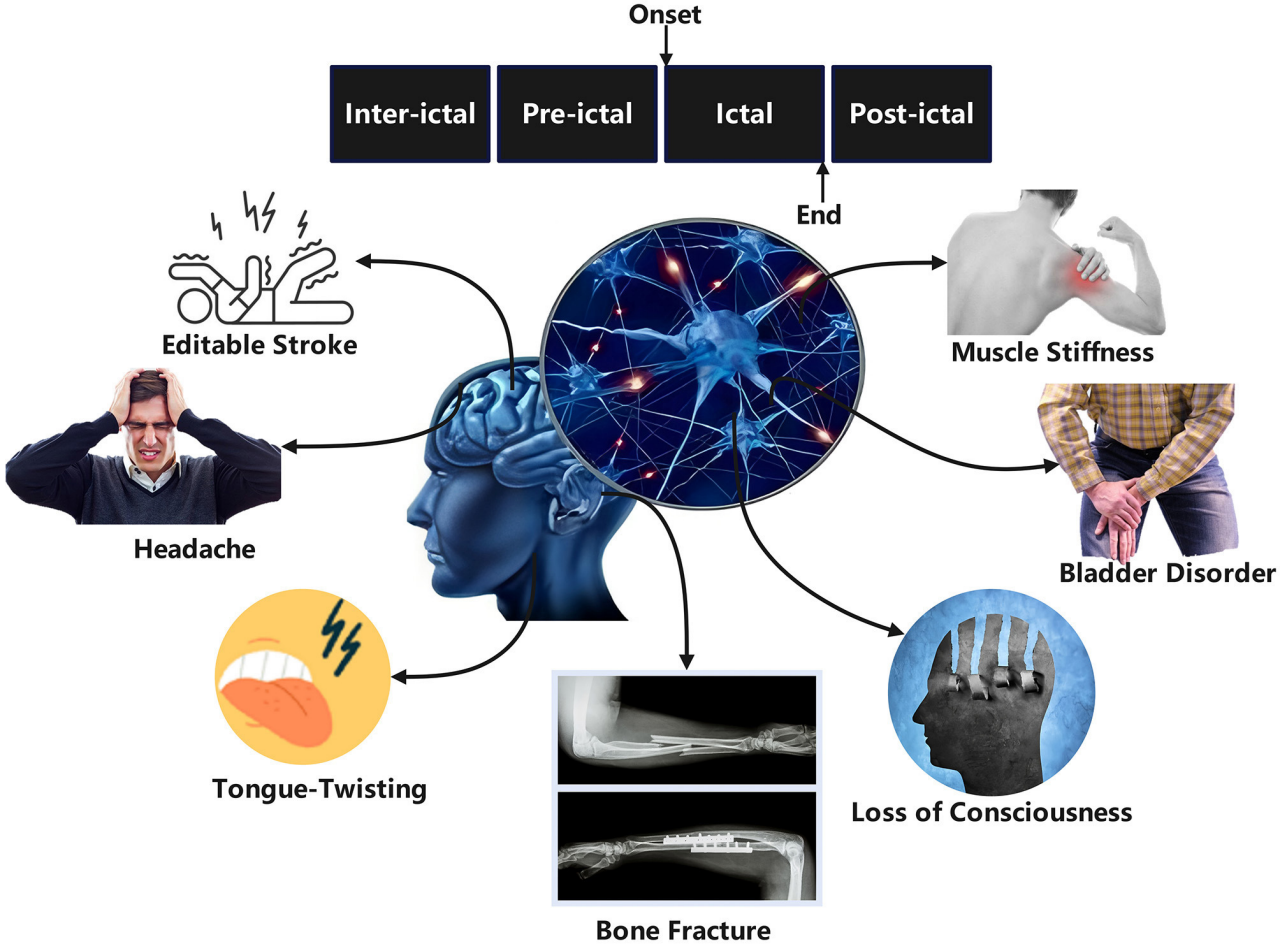
Four different seizure periods and seizure symptoms in epilepsy.

EEG serves as a primary tool for diagnosing and monitoring epilepsy by recording electrical signals generated by neuronal activity in the brain (Nasseri et al., 2024). Numerous studies have focused on extracting various features from EEG signals and integrating them with classifiers to achieve automated seizure detection and characterization (Acharya et al., 2015; Ji et al., 2024; Kantipudi et al., 2024). Based on feature extraction methodologies, three main approaches have emerged: time domain analysis (Solaija et al., 2018; Minasyan et al., 2010), frequency domain analysis (Güler and Übeyli, 2005; Chan et al., 2008), and time-frequency analysis (Niederhauser et al., 2003; Selesnick, 2004; Wang et al., 2018). To better understand the complexity of epileptic EEG analysis, it is essential to examine the different phases of epileptic activity. EEG signals in epilepsy patients can be categorized into four phases: ictal (seizure), preictal (pre-seizure), interictal (between seizures), and postictal (post-seizure) (Usman et al., 2020). Figure 1 illustrates EEG changes during normal brain activity and epileptic seizures in both small and large animals. The ictal phase exhibits clear clinical symptoms and abnormal neuronal discharges, while the preictal phase, characterized by subtle bioelectric abnormalities in specific brain regions minutes before seizure onset, is critical for seizure prediction (Delamont and Walker, 2011). The interictal phase represents the normal state between seizures, and the postictal phase marks the transition from seizure termination to recovery. Figure 2 illustrates the four distinct seizure phases (interictal, preictal, ictal, postictal) and associated symptoms in epilepsy, providing a visual framework for understanding EEG signal classification. Distinguishing preictal from interictal features remains a pivotal research direction in epilepsy prediction. Despite this detailed phase classification, distinguishing preictal from interictal features remains a pivotal research challenge due to the inherent complexity of EEG signals. The nonlinearity, non-stationarity, and complex variability of epileptic EEG signals pose significant challenges to traditional signal processing techniques such as Fourier transform (Tzallas et al., 2009; Li et al., 2016; Shen et al., 2024) and wavelet transform (Mehla and Mehla, 2024; Faust et al., 2015; Hassan et al., 2016; Nishad and Pachori, 2024), limiting their efficacy in feature extraction and pattern recognition (Tzallas et al., 2007, 2009; Lehnertz, 2008). Clinically, preictal and interictal EEG signals are often perceived as indistinguishable, with discernible abnormalities emerging abruptly during the ictal phase (Proix et al., 2018). While automated seizure detection focuses on differentiating ictal from non-ictal EEG signals, prediction aims to distinguish preictal from interictal states (Mirowski et al., 2009; Ibrahim et al., 2022). These technical challenges are compounded by practical limitations in clinical settings. Early detection and prediction of seizures are vital for improving patient quality of life, but the complexity of EEG signals makes these tasks highly challenging (Singh et al., 2021; Abdulkader et al., 2015). Traditional diagnostic reliance on visual interpretation of EEG by clinicians is not only labor-intensive but also prone to subjectivity (Ocak, 2009; Tzallas et al., 2009). These limitations underscore the urgent need for robust automated systems to improve diagnostic efficiency and enable timely interventions.

To address these limitations of traditional linear methods, nonlinear dynamics has emerged as a promising approach, providing novel insights into EEG signal analysis (Lu et al., 2024). Leveraging chaos theory, fractal analysis, and complexity metrics, nonlinear methods such as higher-order spectra (HOS) (Acharya et al., 2012), entropy measures, Lyapunov exponents (Faust et al., 2015), and intrinsic mode functions (IMFs) derived from Empirical Mode Decomposition (EMD) (Fu et al., 2015) have been widely applied to characterize epileptic EEG dynamics. These nonlinear approaches have demonstrated significant advantages in epileptic EEG analysis. For example, (Acharya et al. 2012) demonstrated the efficacy of nonlinear HOS features and entropy in discriminating normal, interictal, and ictal EEG signals. (Darjani and Omranpour 2020) employed phase-space ellipsoid density features for seizure classification, while (Fu et al. 2015) achieved high classification accuracy by combining EMD-derived IMFs with Support Vector Machines (SVMs). (Zeng et al. 2020) further enhanced seizure identification by integrating time-scale decomposition, discrete wavelet transform (DWT), and neural networks.

Although previous reviews have explored nonlinear dynamics in epilepsy detection (Chen et al., 2019; Supriya et al., 2020; Saminu et al., 2023; Acharya et al., 2015; Sharmila, 2018) or prediction (Huang et al., 2025; Iasemidis, 2003; Yadollahpour and Jalilifar, 2015), there remains a gap in systematic mathematical analyses of feature selection mechanisms and model convergence. Compared with previous work, existing reviews are mostly limited to shallow fusion of traditional machine learning frameworks with nonlinear features, while this survey is the first to construct a synergistic analysis paradigm integrating deep learning and nonlinear dynamics under dynamical systems theory. This survey establishes a theoretical framework for distinguishing between detection and prediction through dynamical systems theory: detection focuses on identifying phase transitions using Lyapunov exponent discontinuities, while prediction employs multiscale entropy trend analysis based on ergodic theory. Unlike previous reviews that focus on single technical directions, we clarify the methodological boundaries through a dual-track analytical framework. For feature selection, detection focuses on Lyapunov exponent mutation properties, while prediction emphasizes multiscale entropy trend analysis. For model design, detection models favor CNN time-frequency feature extraction, whereas prediction models favor LSTM long-range dependency modeling. In particular, this survey reveals the advantages of hybrid CNN-LSTM architectures over traditional SVM methods: CNNs adaptively extract Lyapunov exponent spatial gradient features via convolutional kernels, while LSTMs model the evolution of multiscale entropy across time windows through gating mechanisms. These two components synergistically overcome the generalization bottleneck of static feature classifiers. We further analyze the mathematical evolution path from chaos theory models to differential equation-inspired deep learning architectures. In addition, despite the existence of numerous epilepsy databases, new researchers in the field still face challenges in analyzing the impact of chaos theory. To address this, we propose mathematically-based database screening criteria using signal complexity metrics and coverage of dynamical mechanisms to help researchers quickly identify ideal data sources.

The main contributions of this review are summarized as follows. By establishing a comprehensive analysis paradigm of “theoretical mechanism-technical validation-application orientation,” this paper systematically reviews the research progress in automatic detection and prediction of epileptic EEG signals based on nonlinear dynamics, focusing on the extraction mechanisms of core nonlinear characteristics such as Lyapunov exponents and fractal dimensions, as well as their integration and application with hybrid deep learning architectures. Specifically, CNNs can effectively identify spatiotemporal patterns of epileptogenic zones corresponding to Lyapunov exponent mutations through hierarchical extraction of local time-frequency features, while long short-term memory networks (LSTMs) significantly improve the temporal sensitivity for capturing preictal features by modeling the cross-cycle evolution patterns of multiscale entropy. Results demonstrate that by coupling nonlinear dynamical features with deep learning architectures, hybrid models can significantly improve epilepsy detection sensitivity and prediction temporal accuracy. Recent studies further show that Transformer-based attention mechanisms can quantify the coupling strength of nonlinear dynamical features across different brain regions, providing a new paradigm for cross-modal epilepsy detection. However, the field still faces key challenges including noise interference, limited inter-patient generalization capability, complexity of multimodal data fusion, and the sensitivity-specificity trade-off. In particular, the “black box” nature of deep learning models has led to incomplete establishment of interpretable correlations between nonlinear features and clinical pathophysiological mechanisms, which has become a major bottleneck in translating technology into clinical practice. Based on these findings, future research needs to prioritize developing multimodal databases for real-world scenarios to address data heterogeneity, constructing lightweight interpretable models to optimize computational efficiency, and bridging the gap between theoretical innovation and clinical translation through algorithm optimization and standardized evaluation protocols.

### 1.1 Structure of the survey

**Figure 3:**
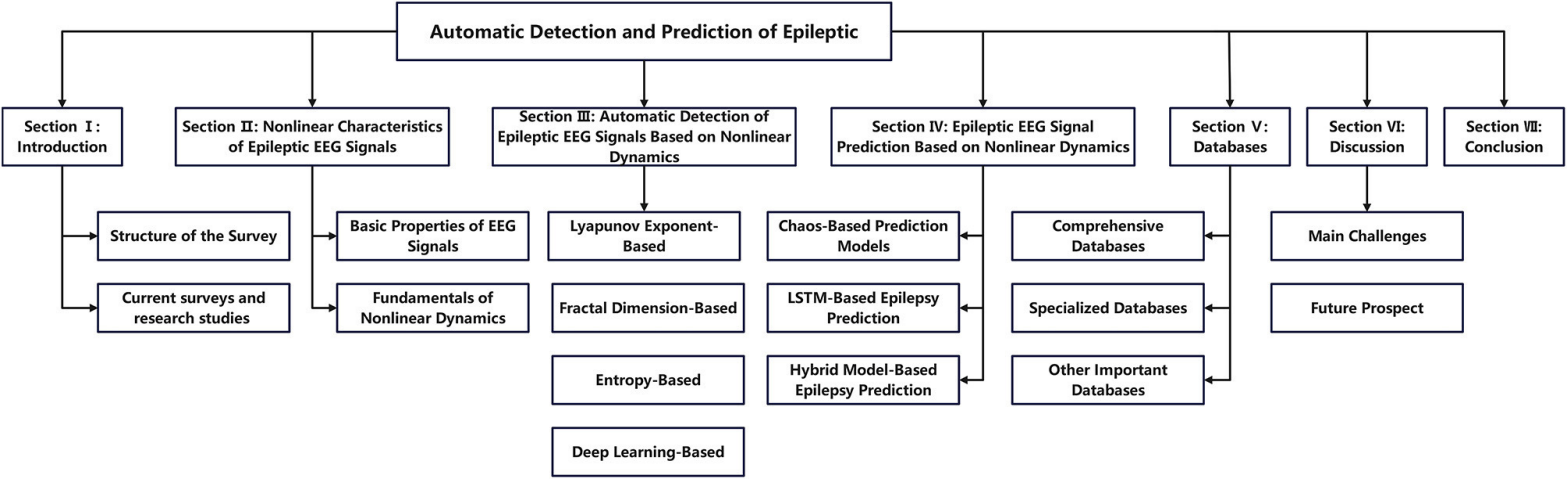
The survey's overview.

As shown in Figure 3, the remainder of this survey is organized as follows. Section 2 introduces the key properties of EEG signals and describes the main types of technologies used and technological innovations. Section 3 presents automatic detection technologies for epileptic EEG signals based on nonlinear dynamics, including methods based on Lyapunov exponents (LE), fractal dimensions (FD), entropy measures, and deep learning approaches. Section 4 introduces epileptic EEG signal prediction models based on nonlinear dynamics, including chaos-based, LSTM-based, and hybrid prediction models. Section 5 provides a comprehensive summary of the main epilepsy databases. Finally, Section 6 discusses the key challenges and future directions in epilepsy detection and prediction research.

## 2 Nonlinear characteristics of epileptic EEG signals

In this section, we describe fundamental properties of epileptic EEG signals and introduce non-linear kinetic parameters commonly used in EEG signal analysis.

### 2.1 Basic properties of EEG signals

EEG is a technique that records the electrical activity of neurons in the cerebral cortex via electrodes, reflecting the synchronized discharge patterns of brain neurons. EEG signals exhibit three fundamental characteristics: non-stationarity, nonlinearity, and multi-scale dynamics (Andrzejak et al., 2012). First, the non-stationarity of EEG signals manifests as temporal variations in their statistical properties, particularly during epileptic seizures, where significant fluctuations occur in both frequency and amplitude (Ahmad et al., 2024). These fluctuations render traditional steady-state analysis methods inadequate for direct application. Second, the nonlinearity of EEG signals arises from the complex interactions among large populations of neurons, which introduce nonlinear effects through synaptic connections and feedback mechanisms (Qi et al., 2024). This results in complex dynamic behaviors that cannot be adequately captured by simple linear models. Finally, the multi-scale nature of EEG signals is evident in their encoding of information across multiple time scales, ranging from millisecond-level fast oscillations to second-level slow wave activities. Consequently, analysis methods must account for dynamic changes at various temporal scales to fully characterize these signals.

### 2.2 Fundamentals of nonlinear dynamics

Nonlinear dynamics establishes rigorous mathematical frameworks for EEG analysis through differential topology and ergodic theory, transcending the limitations of linear methods bound by superposition principles. This mathematical formalism enables phase space reconstruction via Takens' embedding theorem, which provides a diffeomorphic mapping of time series data into higher-dimensional manifolds, preserving the essential dynamics of the underlying system. Fractal dimension calculations employing box-counting algorithms quantify the self-similar geometry of attractors, while entropy measurements derived from the Kolmogorov-Sinai formalism characterize information generation rates in dynamical systems. These methodologies derive their mathematical superiority from the capacity to rigorously quantify critical phenomena. Lyapunov exponents measure exponential divergence of neighboring trajectories in tangent space, correlation dimensions reveal the scaling properties of attractor distributions, Hurst exponents capture long-range dependence through fractional Brownian motion models, and Kolmogorov entropy estimates the rate of information loss. Table 1 summarizes the descriptions, advantages, limitations, and clinical applications of nonlinear dynamics parameters in epileptic EEG analysis. As formalized in Table 1, these parameters constitute functionals of the time series of EEG *X*(*t*), with the maximal Lyapunov exponent λ defined through the limit operator $\lambda =\underset{t\to \infty }{\lim}t^{-1}\ln\;\left(\left||\delta X\left(t\right)||/||\delta X\left(0\right)|\right|\right)$, mathematically encoding the sensitivity of the system to initial conditions. Such constructs transform epileptic transitions into quantifiable instabilities within nonlinear differential equations, bridging abstract dynamical theory with clinical pathophysiology (Vignesh et al., 2025).

**Table 1:** Key nonlinear dynamics parameters for EEG signal analysis: advantages and limitations.

**Parameter/ method**	**Description**	**Advantages**	**Limitations**	**References**
Chaos theory	Analyzes sensitivity to initial conditions and attractor structures to reflect brain dynamics.	Captures nonlinear EEG dynamics. Identifies seizure-related attractor regularity.	Requires high-quality, noise-free data. Computationally intensive for phase space reconstruction.	Müller et al., 2017
Fractal dimension (FD)	Quantifies self-similarity and complexity of signals.	Suitable for analyzing brain signal complexity. Robust to nonstationary data.	Sensitive to parameter selection (e.g., scaling range). Requires long data segments.	Uthayakumar, 2013
Correlation dimension (CD)	Measures geometric complexity of attractors to distinguish chaos from noise.	Discriminates deterministic chaos from stochastic noise. Robust for nonlinear systems.	Computationally heavy for large datasets. Sensitive to embedding parameters.	Grassberger and Procaccia, 1983; Caesarendra et al., 2013
Largest Lyapunov exponent (LLE)	Quantifies divergence rate of nearby trajectories to detect chaotic behavior.	Directly measures chaoticity. Useful for seizure prediction.	Requires high temporal resolution. Noise-sensitive.	Rosenstein et al., 1993
Approximate entropy (ApEn)	Estimates regularity and complexity of time series.	Computationally efficient. Robust to short data and noise.	Biased due to self-counting of patterns. Depends on parameter tuning (e.g., tolerance *r*).	Pincus, 1991
Sample entropy	Improved entropy metric for complexity analysis, avoiding self-matching.	Reduces bias compared to ApEn. Less dependent on data length.	Still sensitive to parameter choices (e.g., *r*, *m*). Computationally slower than ApEn.	Richman and Moorman, 2000
Multiscale entropy (MSE)	Extends Sample Entropy across multiple time scales to evaluate complexity.	Provides multiscale dynamic insights. Detects complexity changes in epilepsy.	Computationally expensive. Requires careful selection of scale factors.	Costa et al., 2005

#### 2.2.1 Chaos theory

Chaos theory is a core concept in nonlinear dynamics. Chaotic systems are extremely sensitive to initial conditions, and even minor changes in initial conditions can lead to significant differences in the long-term behavior of the system, which usually converges to a low-dimensional geometric structure called an attractor, representing the stable state of the system (Sirpal et al., 2025). For a dynamical system:


(1) $$\begin{array}{l} \frac{d\text{x}}{dt}=\text{f}\left(\text{x}\right) \end{array}$$

the attractor is the set *A* in phase space that satisfies **x**(*t*) → **A** when *t* → ∞.

The attractor of EEG signals can reflect the dynamic characteristics of brain activity. EEG signals during epileptic seizures usually exhibit more regular attractor structures, while EEG signals in normal states have more complex attractors (Djemili and Djemili, 2024). By using phase space reconstruction technology, the one-dimensional EEG signal *x*(*t*) is mapped into a high-dimensional phase space to visualize the attractor structure :


(2) $$\begin{array}{l} \text{x}\left(t\right)=\left[x\left(t\right),x\left(t+\tau \right),\ldots ,x\left(t+\left(m-1\right)\tau \right)\right] \end{array}$$

where τ is the time delay, and *m* is the embedding dimension.

#### 2.2.2 Correlation dimension

Correlation dimension (CD) is an important indicator for quantifying the complexity of EEG signals and is a type of fractal dimension (Grassberger and Procaccia, 1983). It can distinguish deterministic chaos from random noise, thereby revealing the underlying dynamic characteristics of the system (Caesarendra et al., 2013). The correlation dimension is usually calculated through the GP algorithm proposed by (Grassberger and Procaccia, 1983), which characterizes the geometric complexity of the attractor in phase space. The correlation dimension is mathematically described as:


(3) $$\begin{array}{l} D_{2}=\underset{\epsilon \to 0}{\lim}\frac{\ln\;\sum_{j=1}^{K\left(\epsilon \right)}p_{j}^{2}}{\ln\;\epsilon } \end{array}$$

where ϵ is the radius of hyperspheres, *K*(ϵ) represents the number of hyperspheres needed to cover the attractor, and *p*_*j*_ is the probability that a trajectory point falls within the *j*-th hypersphere.

#### 2.2.3 The largest Lyapunov exponent

Lyapunov exponents are used to measure the system's sensitivity to initial conditions. A positive Lyapunov exponent indicates that the system exhibits chaotic behavior, while a negative value indicates that the system tends toward stability. In the analysis of epileptic EEG signals, the largest Lyapunov exponent (LLE) is used as a metric to assess the process's dependence on its initial conditions and to quantify the signal's chaotic nature. It defines the rate of divergence of nearby trajectories. The LLE is defined as (Rosenstein et al., 1993):


(4) $$\begin{array}{l} d\left(t\right)=Ke^{c_{1}t} \end{array}$$

where *d*(*t*) represents the average divergence at time *t*, *K* is the initial separation constant, and *c*_1_ represents the LLE that quantifies the exponential divergence rate of neighboring trajectories.

#### 2.2.4 Approximate entropy

Steven Pincus proposed the concept of approximate entropy (ApEn) (Pincus, 1991) to quantify the regularity and complexity of EEG signals. For irregular and complex EEG signals, the ApEn value is higher. ApEn has the advantages of low computational cost, strong robustness to noise, and good adaptability to short data samples. Consider a sequence *X*_*N*_ of length *N*, where *C*_*l*_(*r*) represents the correlation sum for patterns of length *l* within a tolerance *r*. For a given pattern length *l* and tolerance threshold *r*, the approximate entropy ApEn(*X*_*N*_, *l, r*) is defined as:


(5) $$\begin{array}{l} \mathrm{ApEn}\left(X_{N},l,r\right)=\phi \left(l\right)-\phi \left(l+1\right) \end{array}$$

where $\phi \left(l\right)=\frac{1}{N-l+1}\overset{N-l+1}{\underset{i=1}{\sum }}\ln\;C_{i}^{l}\left(r\right)$, and $C_{i}^{l}\left(r\right)$ represents the probability that patterns of length *l* starting at position *i* match within tolerance *r*.

#### 2.2.5 Sample entropy

Sample entropy is an improved version of ApEn, reducing bias. Unlike ApEn, sample entropy assesses the complexity of EEG signals by analyzing the patterns in the signals and does not rely on the measurement of self-similar patterns. Its main advantage is that it is less dependent on the length of the data sample and is more stable in calculation (Skaria and Savithriamma, 2024). Studies have shown that during epileptic seizures, the sample entropy value of EEG signals significantly decreases, indicating an increase in signal predictability (Richman and Moorman, 2000; Valipour et al., 2024; Lin et al., 2025).

#### 2.2.6 Multiscale entropy

Multiscale entropy provides a more comprehensive entropy analysis by calculating sample entropy at different time scales. This method can effectively evaluate the complexity of finite-length EEG signals and reveal the dynamic characteristics of the underlying system at multiple time scales (Costa et al., 2005).

### 2.3 Quantitative characterization of epileptic transition period using nonlinear dynamical indicators

The dynamic changes in EEG signals during epileptic seizures represent a highly nonlinear neurophysiological process that traditional linear analysis methods often fail to characterize adequately. Nonlinear dynamical indicators provide more accurate characterization of critical transitions from pre-ictal to ictal states by quantifying chaotic properties, complexity, and synchronization patterns in EEG signals. During the pre-ictal phase, significant decreases in approximate entropy and sample entropy reveal the gradual loss of complexity in neural electrical activity (Giannakakis et al., 2013; Shen et al., 2013; Song et al., 2012), while declining LLE reflects weakened chaotic characteristics in neural networks (Aarabi and He, 2017). These changes collectively indicate the system's transition toward a highly synchronized ictal state. Simultaneously, the reduction in correlation dimension suggests decreased degrees of freedom in the neural system, and increased phase synchronization index quantifies enhanced functional coupling between brain regions (Lehnertz et al., 2001).

These nonlinear indicators not only detect subtle dynamic changes undetectable by traditional linear methods, but also provide reliable early warning signals minutes to tens of minutes before seizure onset, significantly exceeding the prediction window of linear methods such as power spectral analysis. Crucially, nonlinear indicators can reveal critical slowing phenomena preceding epileptic seizures–decreased system resilience before state transitions–representing sensitivity to stability changes that linear analysis completely lacks. As demonstrated in Table 2, nonlinear indicators substantially outperform traditional linear methods (e.g., power spectral density, band energy) in identifying critical state transitions by capturing the evolution of chaotic properties and complexity in neuronal populations. They achieve superior sensitivity and specificity compared to linear feature-based models, particularly demonstrating enhanced adaptability to interindividual variability and nonstationary EEG signals.

**Table 2:** Comparison of nonlinear and linear indicators for epileptic seizure detection.

**Type**	**Indicator**	**Se (%)**	**Sp (%)**	**Core function**	**Preictal detection advantage**	**References**
**Nonlinear**	Largest Lyapunov exponent (LLE)	86.7	96.7	Characterizes system stability, predicts critical state transition	Nonlinear interdependence index slope >1.5/3 min (8min preictal)	Aarabi and He, 2017
		Phase Synchronization Index	98.6	99.3	Quantifies functional connectivity between brain regions	Time-frequency feature variation rate >1.2/10s (3min preictal)	Gajic et al., 2015
		Multiscale entropy	92.3	89.1	Analyzes signal irregularity across multiple timescales	Scale factor *T* = 6 shows negative correlation (*r* = 4 –0.91) with seizure probability	Kuhlmann et al., 2010
		Correlation dimension (CD)	84.9	93.7	Detects attractor dimension contraction in phase space	Precedes random symptom appearance by 2–5min	Lehnertz et al., 2001
		Sample entropy (SampEn)	95.4	95.8	Measures signal complexity and predictability	Bipolar signal transition rate >3.5/10s (2min preictal)	Shen et al., 2013
		Enhanced sAMPLE eNTROPY	99.0	96.0	Enhanced complexity metric with improved stability	Feature variability >4.2/50 ms (300 ms preictal)	Song et al., 2012
		Approximate entropy (ApEn)	97.3	83.9	Quantifies complexity dynamics in short time series	Decline rate >2.4/100 ms (500 ms preictal)	Giannakakis et al., 2013
**Linear**	Power spectral density (PSD)	72.3	75.8	Detects high-frequency energy anomalies in EEG	N/A	Gotman et al., 1997
		Band energy (delta/theta)	68.9	71.4	Distinguishes interictal/ictal states using frequency bands	N/A	Adeli et al., 2003
		Mean square error (MSE)	N/A	N/A	Evaluates EEG baseline deviation from reference	N/A	Mannan et al., 2018
		Signal-to-artifact ratio (SAR)	N/A	N/A	Quantifies artifact removal effectiveness	N/A	Mannan et al., 2018
		Mutual information	N/A	N/A	Measures statistical dependence between corrected and reference signals	N/A	Ince et al., 2017

Se, sensitivity; Sp, specificity; N/A, not applicable/reported.

## 3 Automatic detection of epileptic EEG signals based on nonlinear dynamics

**Figure 4:**
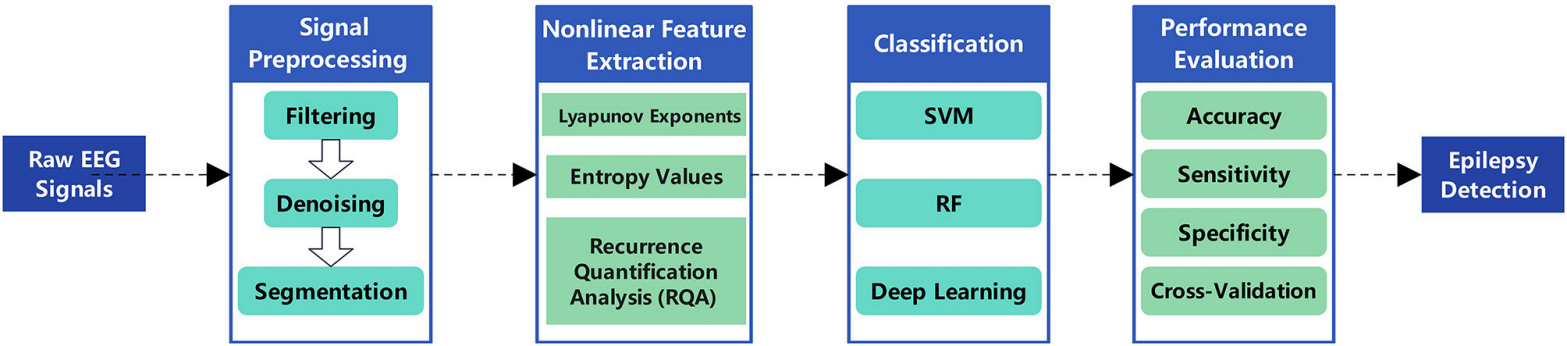
The process of designing an epilepsy detection method.

The primary objective of epileptic seizure detection is to identify seizures that have already occurred from EEG signals. This process typically relies on feature extraction and classification of EEG signals, utilizing nonlinear dynamics methods–such as Lyapunov exponents, entropy measures, and fractal dimensions–to capture significant changes in brain electrical activity during epileptic seizures, thereby effectively distinguishing ictal states from normal brain activity. Classical applications include real-time seizure alarm systems in epilepsy monitoring units and seizure event marking in long-term EEG recordings (Li and Wang, 2024; Benzaid et al., 2024). These systems assist clinicians in rapidly identifying epileptic seizures within clinical environments, improving diagnostic efficiency, and enabling timely medical interventions. The workflow for nonlinear dynamics-based automatic detection of epileptic EEG signals primarily includes signal preprocessing, nonlinear feature extraction, feature selection and dimensionality reduction, classification model construction, and performance evaluation (as shown in Figure 4). Table 3 summarizes the technical features and performance metrics of nonlinear dynamics-based epileptic EEG signal detection methods, providing a systematic comparison for method selection and model optimization in different clinical scenarios.

**Table 3:** Nonlinear dynamics-based methods for epileptic EEG signal detection.

**Method category**	**Approach**	**Key features**	**Techniques used**	**Dataset**	**Performance metrics**	**References**
Lyapunov exponent (LE)	LE-based seizure detection	Quantifies chaotic dynamics via divergence rate in phase space	LLE, SVM	Bonn	High classification accuracy	Iasemidis et al., 1990; Acharya et al., 2013
FD	FD-based complexity analysis	Measures self-similarity and signal irregularity	HFD, PFD, DFA, Line Length (LL)	N/A	High computational efficiency, low error	Janjarasjitt, 2014; Silalahi et al., 2021; Lahmiri, 2018
Entropy analysis	Multi-scale entropy evaluation	Captures signal unpredictability and dynamic transitions	SampEn, PermEn, FuzzyEn, FRFT, DWT, PSD	Bonn, CHB-MIT	Improved accuracy with combined entropy metrics	Oxidative Medicine and Cellular Longevity, 2024; Fei et al., 2017; Zhang J. et al., 2024
Entropy feature fusion	Hybrid entropy frameworks	Integrates entropy with chaos/spectral features	FRFT, FSF, LS-SVM	Bonn	Compact diagnostic tools with high specificity	Zhang et al., 2018; Chen et al., 2019
1D-CNN	Raw EEG signal classification	Direct processing without manual feature extraction	1D-CNN, stacked 1D-CNN	Bonn, CHB-MIT	88.7%–99% accuracy	Ahmedt-Aristizabal et al., 2018; Wang et al., 2021
2D/3D-CNN	Spatial-temporal feature extraction	Enhanced data utilization via signal reshaping and fusion	2D-CNN, 3D-CNN, DFT, DWT	TUH EEG Corpus	Reduced latency (50%)	Avcu et al., 2019; Das et al., 2024; Xu et al., 2024
RNN/LSTM	Temporal dynamics modeling	Long-term dependency capture via gated mechanisms	LSTM, wavelet-LSTM, spectral-LSTM	Freiburg	Improved focal/generalized seizure distinction	Hochreiter and Schmidhuber, 1997; Najafi et al., 2022
Hybrid models	Multi-domain feature fusion	Combines nonlinear dynamics with deep learning	EMD-SVM, FDL (Lime/SHAP), Lyapunov-FD fusion	EPILEPSIAE	Near 100% accuracy, enhanced interpretability	Fu et al., 2015; Khan et al., 2024

### 3.1 Lyapunov exponent-based detection methods

The LE characterizes the chaotic properties of EEG signals by quantifying their sensitivity to initial conditions. Variations in LE reflect dynamic evolutions of neuronal activity. Studies show that Lyapunov exponents of EEG signals decrease significantly during epileptic seizures, indicating enhanced neuronal synchronization and increased system orderliness (Iasemidis et al., 1990). This phenomenon was first identified by Iasemidis et al. in 1990 through the analysis of intracranial EEG (iEEG) signals from epilepsy patients. They observed that the divergence rate of neighboring trajectories in phase space (that is, the maximum Lyapunov exponent, LLE) gradually decreases as seizures approach, signifying a transition from chaotic to ordered brain activity (Iasemidis et al., 1990).

Building on this foundational understanding, Lyapunov exponents have been widely adopted for early seizure detection. (Acharya et al. 2013) combined Lyapunov exponents with support vector machines (SVM) to achieve high classification precision in the Bonn dataset, validating the efficacy of this metric in the analysis of epileptic EEG signals. Recent advances have further enhanced the application of Lyapunov exponents in epilepsy research. (Zhao et al. 2024) pioneered the integration of neurophysiological brain models with nonlinear Kalman filtering, achieving precise detection of focal epileptic seizures through time-evolving Lyapunov spectrum analysis, while revealing that external inputs serve as key drivers of chaotic degree alterations in epileptogenic zones. Complementing this theoretical advancement, (Brari and Belghith 2022) developed a noise-robust PLLE algorithm by refining Wolf's LLE computation method, which effectively preserves essential chaotic signal characteristics while significantly mitigating noise interference, ultimately achieving 100% classification accuracy in epilepsy detection using the Bonn EEG dataset.

### 3.2 Fractal dimension-based detection methods

FD serves as a critical metric for quantifying the complexity and self-similarity of EEG signals, with its value dynamically reflecting alterations in brain states. Research indicates that self-similarity in EEG signals increases markedly during epileptic seizures, with significant differences in FD observed between healthy individuals and patients (Janjarasjitt, 2014; Silalahi et al., 2021; Lahmiri, 2018).

Several computational approaches have been developed to quantify fractal dimensions in EEG analysis. Commonly used computational methods include the Higuchi fractal dimension (HFD), Petrosian fractal dimension (PFD), and detrended fluctuation analysis (DFA). Among these, HFD is preferred for its computational efficiency and low error rates (Silalahi et al., 2021). PFD quantifies signal fluctuation and self-similarity while outperforming fast Fourier transform (FFT) in temporal resolution (Yang et al., 2020). DFA reveals self-similarity and fluctuation trends in non-stationary time series via the Hurst exponent (Lahmiri, 2018). Additionally, line length (LL), a simplified form of FD, exhibits robust performance in detecting the abruptness of seizure-related signals (Ma et al., 2021; Anuragi et al., 2021).

Building upon these established methods, recent developments have further refined fractal dimension analysis for epilepsy detection. (Adda et al. 2025) proposed an enhanced detrended fluctuation analysis (ADFA) method that precisely quantifies long-range temporal correlations (LRTC) and FD in EEG signals. This approach not only validated that FD values during epileptic seizures were significantly higher than during interictal periods (*p* < 0.001), but also achieved a 12.3% improvement in classification accuracy compared to conventional DFA methods, substantially enhancing the detection precision of epileptic seizures through FD analysis.

### 3.3 Entropy-based detection methods

Entropy, a vital metric for quantifying signal irregularity and unpredictability, encompasses common variants such as approximate entropy (ApEn), sample entropy (SampEn) and multiscale entropy (MSE). In epileptic EEG signal analysis, entropy variations reflect dynamic signal properties. Studies reveal significant reductions in entropy during epileptic seizures, indicating increased signal predictability.

Early approaches to entropy-based epilepsy detection focused on combining different entropy measures. Oxidative Medicine and Cellular Longevity (2024) used entropy-based features—including sample entropy (SampEn), permutation entropy (PermEn), and fuzzy entropy (FuzzyEn)—individually and in combination to form three-dimensional feature vectors. Their findings demonstrate that combining SampEn, PermEn, and FuzzyEn yields the highest accuracy and recall rates. However, this approach lacks integration of temporal or frequency-domain information. To address this limitation, (Fei et al. 2017) developed a more comprehensive approach by adopting the fractional Fourier transform (FrFT), adaptive maximum Lyapunov exponents and energy characteristics to capture chaotic and frequency domain characteristics of epileptic EEG signals. Similarly, Zhang J. et al. (2024) adopted a more comprehensive methodology by extracting features via the discrete wavelet transform (DWT), power spectral density (PSD), standard deviation, band energy and fuzzy entropy. While this method effectively aggregated diverse feature information, it also introduced redundancy and increased computational complexity.

Recognizing the need for feature optimization, (Zhang et al. 2018) generated 2,794 features per sample using multiple extraction techniques, which were subsequently evaluated through sequential selection algorithms (IE, VARA, IRFE, and BackF) to filter critical features. Building on these multi-feature approaches, (Chen et al. 2019) used eight entropy algorithms, DWT-based ANOVA, FSF, and LS-SVM to differentiate ictal and interictal EEG states, ultimately providing a compact seizure diagnostic tool with exceptional classification precision, sensitivity, and specificity.

### 3.4 Deep learning-based nonlinear feature fusion methods

Deep learning, a key branch of machine learning, has gained prominence in the detection of epileptic EEG signals, with convolutional neural networks (CNNs) emerging as the dominant architecture due to their automated feature extraction capabilities. In the early development of CNN-based approaches, (Ahmedt-Aristizabal et al. 2018) pioneered the use of one-dimensional CNN (1D-CNN) for the direct classification of raw EEG signals, achieving an average accuracy of 88.7%. Their design eliminates manual feature extraction, substantially streamlining workflows. Building upon this foundation, (Wang et al. 2021) further employed stacked 1D-CNN architectures to achieve over 99% accuracy on two public datasets, though concerns about the reliability of the results arose due to missing test sets.

To enhance data utilization efficiency beyond 1D approaches, two-dimensional CNNs (2D-CNNs) have been extensively explored. (Avcu et al. 2019) surpassed traditional multichannel models using only dual-channel data. Similarly, (Das et al. 2024) validated the superiority of 2D-CNNs through the reshaping of EEG data format. Addressing the challenge of limited data availability, (Pan et al. 2022) developed a lightweight 2D-CNN that integrates the discrete Fourier transform (DFT) and the discrete wavelet transform (DWT), demonstrating strong performance under limited data, although insufficient testing hindered generalization. Advancing further into higher-dimensional architectures, the introduction of three-dimensional CNNs (3D-CNNs) has pushed technical frontiers: (Xu et al. 2024) reduced detection latency by 50% through probabilistic forecasting, significantly improving real-time capabilities.

Beyond CNNs, the exploration of alternative deep learning models is progressing. Recognizing the temporal characteristics of EEG signals, recurrent neural networks (RNNs) and their variants, long-short-term memory networks (LSTMs), demonstrate unique advantages. LSTMs address the vanishing gradient problem in traditional RNNs through gating mechanisms, making them effective tools for processing long-sequence data (Hochreiter and Schmidhuber, 1997). (Najafi et al. 2022) improved the classification accuracy for focal and generalized epilepsy by combining wavelet transforms with LSTM-RNNs. Extending this approach, (Singh and Malhotra 2022) constructed a dual layer LSTM network based on spectral characteristics, verifying performance improvements through complex architectures. Further integrating feature engineering with LSTM architectures, (Goel et al. 2023) optimized the diagnostic efficacy by integrating entropy characteristics with LSTMs. However, these methods focus primarily on frequency domain and temporal information, neglecting the spatial dimensions of EEG signals and limiting the comprehensive utilization of multidimensional features.

While mainstream approaches have focused on established architectures, applications of generative adversarial networks (GANs) and transfer learning remain exploratory, but show promise. (Truong et al. 2019) pioneered GANs for epilepsy prediction, opening new avenues beyond data augmentation. Meanwhile, multimodal approaches, though enabling real-time detection, face hardware, and data limitations. In pursuit of biologically-inspired and energy-efficient solutions, spiking neural networks (SNNs), which mimic biological neuronal dynamics for low-energy processing, have seen training challenges addressed by (Zhang et al. 2025) through pulsed recurrent neural networks. Addressing computational efficiency concerns, (Liu et al. 2017) reduced memory costs by 75% using cosine convolutional networks (COSCNN). Additionally, the capsule networks of (Sabour et al. 2017) overcome CNN limitations in the modeling of feature relationships. These innovations drive models toward efficiency and interpretability.

Recent breakthroughs are primarily hinged on the integration of deep learning with non-linear dynamics features. (Fu et al. 2015) achieved high classification accuracy by combining intrinsic mode functions (IMF) derived from empirical mode decomposition (EMD) with SVMs. Advancing interpretability alongside accuracy, (Khan et al. 2024) proposed a fuzzy deep learning (FDL) framework that integrates locally interpretable models (LIME) and Shapley values (SHAP), improving both accuracy and interpretability. Demonstrating the power of feature fusion, Acharya et al. attained near-perfect classification accuracy on the Bonn data set by combining Lyapunov exponents and fractal dimensions. These studies demonstrate that multidimensional feature fusion comprehensively captures dynamic EEG characteristics, providing clinically reliable insights.

## 4 Epileptic EEG signal prediction based on nonlinear dynamics

**Figure 5:**
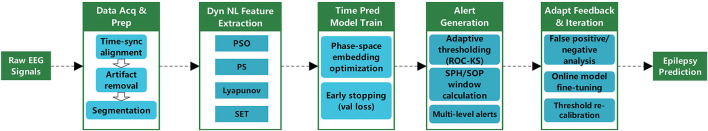
The process of designing an epilepsy prediction method.

Clinical manifestations of epileptic seizures can be categorized into three distinct patterns: abrupt seizures that occur against normal background activity, reflex epilepsy triggered by external stimuli, and progressive transitions from normal activity to seizures through precursor states (Mormann et al., 2007). The framework based on non-linear dynamics for epilepsy prediction integrates essential components including signal preprocessing, feature extraction, model construction, and performance evaluation as illustrated in Figure 5. Despite extensive research spanning several decades, seizure prediction remains a formidable challenge. Studies reveal that specific non-linear dynamic alterations, including enhanced phase synchronization and anomalous complex network properties, occur in EEG signals minutes to hours before seizures (Cook et al., 2013; Kuhlmann et al., 2018). Although intracranial EEG demonstrates superior preictal predictive capabilities compared to scalp EEG, its clinical utility remains restricted by procedural invasiveness.

The central objective of epilepsy prediction is to identify subtle preictal biomarkers in EEG signals to enable timely warnings; the main technical challenge lies in distinguishing dynamic variations between preictal and interictal states. Nonlinear dynamics methodologies such as phase synchronization analysis and complex network modeling, when integrated with machine learning algorithms like support vector machines and random forests or deep learning architectures including convolutional and long-short-term memory networks, effectively detect transitional preictal states. Notable applications include intelligent implantable devices that reduce seizure frequency through real-time EEG monitoring and targeted interventions such as electrical stimulation. Additionally, predictive technologies refine pharmacological regimens to minimize adverse effects. Table 4 summarizes key methodologies, model architectures, performance metrics, and associated references for chaotic, LSTM-based, and hybrid models in epileptic seizure prediction.

**Table 4:** Summary of nonlinear dynamics and machine learning models for epileptic seizure prediction.

**Model**	**Type**	**Key methods/features**	**Performance metrics**			**References**
			**AUC**	**F1**	**Sen./Spe**.	
PSR with PSO	Chaotic	Adaptive parameter optimization, Lyapunov exponent analysis	88%	N.R.	85%/78%	Lu et al., 2024
PSR + PS + SET	Chaotic	Combines PSR, PS, and state space entropy for nonlinear feature detection	99%	93%	99%/88%	Lu et al., 2024
2L-LSTM with FFT	LSTM	Spectral power and amplitude features via FFT, two-layer LSTM	94%	89%	98%/88%	Singh and Malhotra, 2022
LSTM + DWT	LSTM	Integrates discrete wavelet transform and statistical moments	N.R.	N.R.	92%/85%	Tsiouris et al., 2018
CNN-LSTM hybrid	LSTM	CNN extracts spatial features, LSTM models temporal dependencies	81%	74%	77%/74%	Lu et al., 2022
CNN-RQA	Hybrid	Combines CNN for local features and RQA for recurrence analysis	92%	N.R.	88%/N.R.	Sunaryono et al., 2022
EMD-GRU	Hybrid	EMD decomposes EEG into IMFs; GRU captures temporal dynamics	N.R.	94%	N.R.	Ma et al., 2023
CNN-GRU-AM	Hybrid	Discrete wavelet features + attention mechanism for feature weighting	95%	N.R.	95%/94%	Zhang J. et al., 2024
Transformer-LSTM	Hybrid	Focuses on key brain regions via attention mechanism	N.R.	87%	N.R.	de Santana Correia and Colombini, 2022
SE-TCN-BiGRU	Hybrid	Channel selection + TCN-BiGRU for lightweight design	N.R.	N.R.	N.R.	Zhu et al., 2024
LMA-EEGNet	Hybrid	Lightweight multi-branch network for EEG signal processing	N.R.	N.R.	N.R.	Zhou et al., 2024
EEG + fNIRS	Cross-Modal	Combines EEG and near-infrared spectroscopy	N.R.	N.R.	89%/95%	Sirpal et al., 2019
EEG + rs-fMRI	Cross-Modal	Merges resting-state fMRI with EEG for seizure focus localization	N.R.	N.R.	96%/97%	Hosseini et al., 2020

N.R., not reported in the original study.

### 4.1 Chaos-based prediction models for epilepsy

Chaos-based prediction models analyze nonlinear dynamic properties within EEG signals—such as phase space reconstruction and Lyapunov exponent variations—to identify critical state transitions preceding seizures. Phase space reconstruction techniques, for instance, employ embedding dimensions and time delay parameters to construct multidimensional phase spaces, enabling the tracking of attractor structure dynamics. Research indicates a significant reduction in attractor dimensionality within EEG signals during preictal phases, reflecting heightened neuronal synchronization.

To enhance model robustness and adaptability, recent advancements incorporate adaptive parameter optimization algorithms. Specifically, particle swarm optimization-enhanced phase space reconstruction dynamically adjusts embedding dimensions to improve noise resilience. Clinically, these models have enabled long-term pediatric epilepsy monitoring, achieving seizure predictions 10–15 min in advance with sensitivities surpassing 85%. However, chaos models remain constrained by data length dependencies, necessitating sliding window strategies to enhance real-time performance. Building on these foundational chaos-based approaches, (Lu et al. 2024) systematically applied phase space reconstruction, phase synchronization, and state space entropy to investigate nonlinear characteristic disparities in epileptic EEG signals. Their work identifies distinct preictal nonlinear features compared to ictal and interictal states, substantially advancing seizure detection and prediction efficacy.

### 4.2 LSTM-based epilepsy prediction

Long short-term memory (LSTM) networks leverage their gating mechanisms and temporal modeling proficiency to capture long-term dependencies in EEG signals during transitions from normal to epileptic states, solidifying their role as a cornerstone of seizure prediction. In pioneering applications of LSTM to epilepsy prediction, (Tsiouris et al. 2018) first demonstrated LSTM's potential on the CHB-MIT dataset, achieving prediction sensitivities and specificities exceeding 99% and outperforming conventional convolutional neural networks. Building upon this foundational work, subsequent studies expanded the utility of LSTM: (Singh and Malhotra 2022) fused fast Fourier transform-derived spectral power and mean spectral amplitude features to develop a dual-layer LSTM network, improving accuracy through multidimensional feature integration. Similarly, (Tsiouris et al. 2018) incorporated statistical moments, time domain features, and discrete wavelet transforms within an analogous framework, empirically validating LSTM robustness in multimodal feature processing.

Recognizing the potential benefits of combining different architectural strengths, researchers are increasingly adopting hybrid designs. (Lu et al. 2022) proposed an end-to-end multiframe network that combined convolutional neural networks for spatial feature extraction with LSTMs for temporal dependency modeling, surpassing standalone models on both scalp and intracranial EEG data. As the field matured, addressing the interpretability of these complex models has gained prominence. (Gao et al. 2023) devised a multiscale prototype-part network that delivers transparent inference through self-explanatory mechanisms, maintaining state-of-the-art performance while resolving deep learning opacity challenges and providing clinically actionable insights. Advancing hybrid architectures further, (Alharbi et al. 2024) developed a hybrid graph neural network architecture based on Bayesian optimization, which effectively captures spatiotemporal features of EEG signals through CNN-LSTM integration with adaptive skip connections, significantly enhancing the predictive performance of LSTM on imbalanced epilepsy datasets.

Moving toward practical clinical implementation, current research emphasizes LSTM lightweighting and real-time optimization. Architectures integrating multiscale entropy analyze entropy decay patterns across temporal scales to predict seizures 20 min in advance with specificities exceeding 90%. Complementing these temporal approaches, attention-enhanced Transformer-LSTM models prioritize abnormal discharges in critical brain regions such as the temporal and frontal lobes, reducing false positives. Looking ahead, future investigations must prioritize lightweight LSTM deployment in wearable devices while advancing multimodal feature fusion and biologically inspired architectures to improve clinical applicability.

### 4.3 Hybrid model-based epilepsy prediction

Hybrid models unify non-linear dynamics features with deep learning architectures to harmonize interpretability and predictive performance. A representative example is the CNN-RQA hybrid model, where convolutional neural networks extract localized time-frequency EEG features while recurrence quantification analysis generates dynamic metrics including recurrence rate and laminarity. Combined features are subsequently processed by fully connected layers for prediction. Using the Freiburg data set, this model detects seizures 25 min in advance, demonstrating superior sensitivity to low-frequency oscillatory signals compared to standalone approaches (Sunaryono et al., 2022). Expanding upon this CNN-based approach, alternative hybrid methodologies integrate empirical mode decomposition with gated recurrent units, decomposing EEG signals into intrinsic mode functions for multiresolution nonlinear feature extraction followed by temporal dependency modeling, achieving exceptional precision in the prediction of neonatal seizures (Ma et al., 2023). Building on these foundational hybrid designs, Zhang J. et al. (2024) further advanced this paradigm through a CNN-GRU-AM model employing discrete wavelet transform-based feature extraction and gated attention mechanisms to enhance detection precision.

Addressing the computational challenges inherent in processing long EEG sequences, hybrid models increasingly integrate attention mechanisms and residual networks. The Transformer architecture manages extended EEG sequences efficiently through multi-head attention, reducing temporal complexity (de Santana Correia and Colombini, 2022; Rukhsar and Tiwari, 2023). Simultaneously, residual networks mitigate gradient vanishing through skip connections, enhancing model stability (He et al., 2016). Implementing these architectural advances, (Huang et al. 2024) introduced a temporal convolutional self-attention network for extracting autonomous features, while (Zhu et al. 2024) proposed a SE-TCN-BiGRU model combining channel selection with parameter efficiency. Focusing on real-world deployment constraints, (Zhou et al. 2024) streamlined complexity through a lightweight multi-branch network tailored for real-time monitoring.

Beyond single-modality approaches, multimodal data fusion has emerged as a pivotal factor in improving detection robustness. (Ahmedt-Aristizabal et al. 2018) integrated facial, postural, and EEG features to differentiate mesial and lateral temporal lobe epilepsy. Demonstrating the benefits of physiological signal integration, (Sirpal et al. 2019) combined EEG with functional near-infrared spectroscopy, increasing classification accuracy by 8%. Advancing toward comprehensive brain imaging fusion, (Hosseini et al. 2020) fused resting-state fMRI and EEG for the localization of the epileptogenic zone by edge computing, while (Martini et al. 2021) optimized real-time sensitivity using stereo-EEG and video-EEG with dynamic thresholds.

While these technological advances show considerable promise, generalizability remains a critical challenge that requires rigorous validation. (Aslam et al. 2022) reported exceptional CNN-LSTM performance on specific data sets, but noted variability between data sets. Similarly, Zhang X. et al. (2024) reduced computational demands through temporal attention models, but retained dependency on high-quality annotations. To address these limitations, future efforts must prioritize multicenter validation and explore few-shot learning alongside unsupervised strategies to accelerate clinical translation.

## 5 Databases

In nonlinear dynamics-driven automated detection and prediction research for epileptic EEG signals, the construction of high-quality databases forms the core foundation for algorithm development and validation. In this section, we systematically review the current internationally recognized epileptic EEG database systems, categorizing them into three major classes based on data characteristics and application scenarios. As systematically outlined in Table 5, the technical specifications and application scenarios of these databases provide quantitative criteria to select the appropriate data resources in nonlinear dynamics-driven epileptic analysis.

**Table 5:** Comparative technical specifications of epileptic EEG databases by category and key parameters.

**Database**	**Category**	**Subjects**	**Duration/ size**	**Recording type**	**Sampling rate**	**Key features**	**Limitations/clinical gaps**	**References**
Siena scalp EEG database v1.0.0	Comprehensive	14 patients	128 h	Scalp + EKG	512 Hz	47 video-verified seizures; < 0.5s annotation precision	Limited patient diversity; no intracranial data	Detti, 2020
CHB-MIT scalp EEG database	Comprehensive	23 pediatric	>850 h	Scalp	256 Hz	Drug-free longitudinal data; 162 seizure events	Lacks preictal annotations; pediatric-only cohort	Goldberger et al., 2000
EPILEPSIAE database	Comprehensive	275 patients	>45,000 h	Scalp + Intracranial	200–5000 Hz	2,662 seizures; MRI integration; multi-modal clinical phenotypes	Heterogeneous recording protocols across centers	Ihle et al., 2012; Klatt et al., 2012
Freiburg Seizure Prediction Project EEG	Specialized	21 patients	Pre/post-ictal recordings	Intracranial	256 Hz	128-channel coverage; 50-min pre-ictal data	Small sample size; no scalp EEG for comparison	Ihle et al., 2012
Temple University Hospital EEG Corpus	Specialized	N/A	>50 TB (26,846 records)	Scalp	200–5,000 Hz	Three-tier quality control; 37.2% abnormal EEG	Unclear seizure annotation methodology	Ochal et al., 2020
Bonn EEG database	Other	5 groups	100 segments/group	Scalp	173.61 Hz	Controlled ictal/interictal partitions; single-channel 23.6s segments	Artificial segmentation; single-channel limitation	Andrzejak et al., 2001
UPenn-Mayo seizure detection challenge	Other	Cross-species	1:24 ictal/interictal ratio	Mixed	Variable	Double-blind annotations; multi-scale performance metrics (sensitivity/specificity/latency)	Limited clinical applicability due to animal data	Temko et al., 2015

### 5.1 Comprehensive databases

Comprehensive databases are characterized by multimodal data integration and complete clinical information, providing a multidimensional analytical basis for nonlinear dynamics modeling. A prime example is the Siena Scalp EEG Database v1.0.0 (Detti, 2020), which integrates long-term monitoring data (128 h from 14 patients with refractory epilepsy, synchronized acquisition of dual-channel EKG signals using a high sampling rate of 512 Hz, and contains 47 video-verified seizure events. Its significant advantage is that it provides accurate annotations of the onset and end time of seizures (error < 0.5 s), suitable for the time-frequency characterization of seizure detection algorithms.

Another prominent comprehensive database is the CHB-MIT database,^1^ which contains continuous scalp EEG recordings (total duration >850 h) from 23 pediatric patients at Children's Hospital Boston. Data were collected using a drug-free clinical monitoring protocol and contained 162 definite seizures (Goldberger et al., 2000). Its characterization of longitudinal data provides important support for the study of cyclical seizure patterns.

The most comprehensive and ambitious database in this category is EPILEPSIAE (Ihle et al., 2012), which is one of the most comprehensive epilepsy databases available. As the flagship epilepsy research platform of the European Union, this repository integrates intracranial/scalp synchronized EEG data from 275 patients (>45,000 h of total recordings), covering 2,662 seizure events and corresponding MRI images (Klatt et al., 2012). Its innovation lies in the construction of a multidimensional data correlation system (electrophysiology-imaging-clinical phenotype), which supports the study of epilepsy network dynamics based on complex systems theory.

### 5.2 Specialized databases

In contrast to comprehensive databases, specialized databases, on the other hand, focus on specific types of EEG signals or specific research questions related to epilepsy. These databases typically provide highly specialized data that are applicable to specific research needs.

The premier example of specialized databases is the Freiburg Seizure Prediction Project EEG Database,^2^ which provides data from the Freiburg Seizure Prediction Project through long-term intracranial electrode monitoring (sampling rate 256 Hz) in 21 patients, providing continuous recordings from 50 min before to 30 min after seizure onset (Ihle et al., 2012). Its high spatiotemporal resolution feature (covering 128 deep cortical electrode channels) is particularly suitable for the extraction of non-linear features in the seizure onset zone.

Complementing intracranial data with large-scale clinical recordings, Temple University Hospital EEG Resources (Temple University EEG Corpus—Downloads) contains 26,846 clinical EEG recordings collected between 2002 and 2017 (total data volume >50 TB) using a three-tier quality assessment system (Level I: signal integrity; Level II: clinical diagnostic consistency; Level III: accuracy of event annotation) (Ochal et al., 2020). Its large-scale abnormal EEG samples (37.2% of the total) provide important training resources to build robust seizure detection models.

### 5.3 Other important databases

In addition to the aforementioned databases, there are several other significant databases that serve specific research purposes, such as the Bonn EEG Database (Andrzejak et al., 2001) and the Seizure Detection Challenge database from the UPenn and Mayo Clinics (Temko et al., 2015).

The Bonn EEG Database represents a foundational resource for controlled epilepsy research, comprising five distinct datasets: Healthy Group A/B, Interictal Group C/D, and Seizure Group E. Each data set includes 100 segments of single channel EEG recordings lasting 23.6 s, sampled at 173.61 Hz. The database's rigorously controlled experimental conditions, including open/closed eye states and a clear distinction between seizure periods and interictal intervals, make it an ideal platform for validating the stability of nonlinear features, such as Lyapunov exponents and entropy values.

Extending beyond human subjects to include animal models, the Seizure Detection Challenge database from the UPenn and Mayo Clinics provides data from human and canine epilepsy models, with a seizure-to-interictal time ratio of 1:24. This database employs a double-blind annotation strategy, validated by three independent experts, and incorporates a multiscale evaluation framework that assesses sensitivity, specificity, and delay time to enhance the clinical utility of seizure detection algorithms.

## 6 Discussion

Mathematical formulations for the detection and prediction of epilepsy require rigorous analysis within a probabilistic framework. Detection tasks are defined by classification metrics (accuracy, sensitivity, specificity) within a probabilistic space, while predictive models involve stochastic processes in Banach spaces. The core of this distinction lies in the inherent ambiguity of epilepsy prediction criteria, such as the maximum false positive rate and seizure prediction window, which are defined through Lebesgue integration on nonstationary time intervals. When the time domain is ill-defined, it leads to σ-algebra incompleteness. The Maiwald criteria for epilepsy prediction, including the maximum false positive rate (FPRmax), the seizure prediction horizon (SPH), and the seizure occurrence period (SOP), are widely adopted (Ratcliffe et al., 2024). However, there is still debate about the precise definitions of these key metrics. For instance, the calculation of FPR depends on the time interval. Longer intervals may underestimate the false positive rate, complicating direct comparisons between studies. Most predictive models currently use clinical time windows, with SPH ranging from 1 to 15 min and SOP from 30 min to several hours. Nevertheless, these parameters impose Lipschitz continuity constraints on the predictive calculus, potentially violating the ergodic properties governed by chaotic attractors in EEG dynamics, thereby limiting algorithm performance in dynamic environments.

Data partitioning strategies can have a measurable impact on model generalization, as validated through the Kolmogorov-Smirnov test. Preprocessing methods form an orthogonal basis in Hilbert space–wavelet transforms achieve multi-resolution analysis through Daubechies scale functions, while EMD performs adaptive decomposition through sifting processes. Current research typically employs patient-independent data partitioning strategies, with few studies designed based on patient specificity, but the lack of transparency in data partitioning methods often leads to reduced comparability of results. In terms of data augmentation, generative adversarial networks (GANs) can produce synthetic signals, albeit at high computational cost, while lightweight methods like window overlapping can expand the dataset and enhance model robustness. Training on multiple datasets can improve model generalization, but consistency across different data formats and annotation standards needs to be addressed.

The mathematical significance of the CHB-MIT dataset lies in its tensor structure (channels × time × patients), which can be analyzed through Tucker decomposition. It is characterized by its large scale, flexible structure, and continuous recording of interictal and preictal EEG signals, and has become the most widely used public dataset in epilepsy detection and prediction research (Wei et al., 2018). Its patient-grouped storage mechanism facilitates the adoption of patient-specific and cross-patient data partitioning strategies, while allowing for customizable time windows to define ictal and interictal periods, thus providing great flexibility for model training. However, its laboratory samples lack isomorphic mappings to real-world dynamic mechanisms, which can lead to topological anomalies in clinical applications (Bhagubai et al., 2024). Moreover, existing public datasets often lack systematic annotations of preictal signals, thus limiting the generalization capability of predictive models.

### 6.1 Main challenges in current research

Despite significant advances in the automatic detection and prediction of epileptic EEG signals using nonlinear dynamics, their clinical translation still faces fundamental mathematical obstacles rooted in the incompatibility of probabilistic frameworks and topological data constraints. First, data quality and generalizability remain core bottlenecks. EEG signals are highly susceptible to environmental noise and physiological artifacts. The divergence of patient-specific EEG distributions violates the Lipschitz continuity assumption in the reproducing kernel Hilbert space, leading to exponential growth of the generalization error bounds with distributional bias and significantly degrading model performance across patients or devices. Furthermore, although existing public datasets are large, their controlled laboratory environments differ from real-world dynamics, leading to topological distortions in the feature manifolds and limiting clinical applicability, thus necessitating technical adjustments for clinical deployment (Jemal et al., 2021). Second, the complexity of multimodal data fusion hinders a complete understanding of epileptic pathophysiology (Rai et al., 2025). Insufficient temporal alignment and feature integration of multimodal data currently lack standardized protocols, thus increasing the complexity of model design.

Existing algorithms face challenges in balancing sensitivity and specificity in epileptic prediction tasks. As the seizure prediction window (SOP) expands, the increase in false positive rates (FPR) becomes a non-negligible issue, while the trade-off between precision and recall further limits model practicality. To address these limitations, research should focus on end-to-end process optimization. In the preprocessing stage, Butterworth filters or wavelet packet transforms (WPTs) can effectively remove noise and improve signal-to-noise ratios. Feature engineering needs to retain classic nonlinear features like entropy and correlation dimensions while exploring new features with high inter-class variance, using recursive feature elimination (RFE) or support vector machine recursive feature elimination (SVM-RFE) to select the optimal feature subset, reducing redundancy and improving computational efficiency. In classifier optimization, prioritizing hyperparameter tuning of mature models like SVMs and CNNs, and combining with statistical validation to optimize decision thresholds, rather than blindly developing new architectures, can more effectively enhance performance.

Feature extraction and selection are the core stages of model optimization. (Jemal et al. 2021) found through analyzing the false positive rate and overlap rate that a specific combination of features can significantly reduce the algorithm's dependence on data volume and maintain robustness in handling small samples. (Zhang et al. 2018) used variance analysis (VarA) and backward feature selection (BackFS) to eliminate features with low discrimination, significantly improving classification accuracy. (Savadkoohi et al. 2020) combined *t*-tests with sequential forward floating search (SFFS) to select the optimal feature set, ultimately achieving 100% precision, sensitivity, and specificity. These cases demonstrate that refined feature engineering can effectively balance model complexity and performance, providing a viable path for algorithm improvement. However, extracting Morse-Smale complexes from EEG fluctuations remains an unsolved problem in geometric analysis.

### 6.2 Future prospect

#### 6.2.1 Multimodal data integration and the development of novel analysis methods represent the primary avenues for advancing epilepsy research

Combining EEG with multiple physiological signals, such as electrocardiogram (Slater et al., 2024), electromyogram (Wu, 2024), and oxygen saturation (Wertheim et al., 2024), enables comprehensive characterization of the physiological correlates of epileptic seizures. The introduction of advanced nonlinear dynamic techniques, such as complex network analysis and topological data mining, is anticipated to enhance the robustness of feature representation. Furthermore, interdisciplinary collaboration will deepen the understanding of epilepsy's pathophysiological mechanisms and provide biologically inspired insights for algorithm design. For instance, (Teixeira et al. 2014) identified circadian rhythm patterns in frontal lobe epilepsy, highlighting the importance of exploring the relationship between sleep characteristics and seizure timing, which could potentially lead to the development of time-segmented adaptive classifier combination strategies.

#### 6.2.2 Optimizing algorithms and constructing standardized evaluation frameworks constitute another core task

Current research is constrained by dataset singularity, homogenized preprocessing approaches, and the absence of unified evaluation criteria. Public datasets are predominantly derived from controlled environments and fail to capture the complexity of real-world scenarios. Definitions of key metrics, such as false positive rate (FPR) and seizure prediction horizon (SPH), lack consensus, resulting in limited comparability across studies (Teixeira et al., 2014). In the future, it will be essential to establish a multi-center database encompassing diverse epilepsy types, age groups, and medication backgrounds, alongside standardized evaluation protocols. At the algorithmic level, enhancing discrimination between inter-ictal and pre-ictal phases through dual-threshold segmentation based on temporal and feature dimensions, or employing lightweight models to reduce computational burden while meeting real-time monitoring requirements, will be critical.

#### 6.2.3 Personalized medicine and clinical translation represent the ultimate objectives

Developing customized models that account for individual patient differences and leveraging explainable AI technologies can bolster clinical trust. Optimizing embedded algorithms within intelligent wearable devices will facilitate the widespread adoption of home-based monitoring solutions. Simultaneously, addressing regulatory compliance and ethical considerations will ensure that technological implementations adhere to medical standards. Only through continuous technological innovation and cross-domain collaboration can the seamless transition of epilepsy prediction from laboratory settings to clinical practice be achieved, thereby providing patients with precise and timely intervention plans.

**Figure 6:**
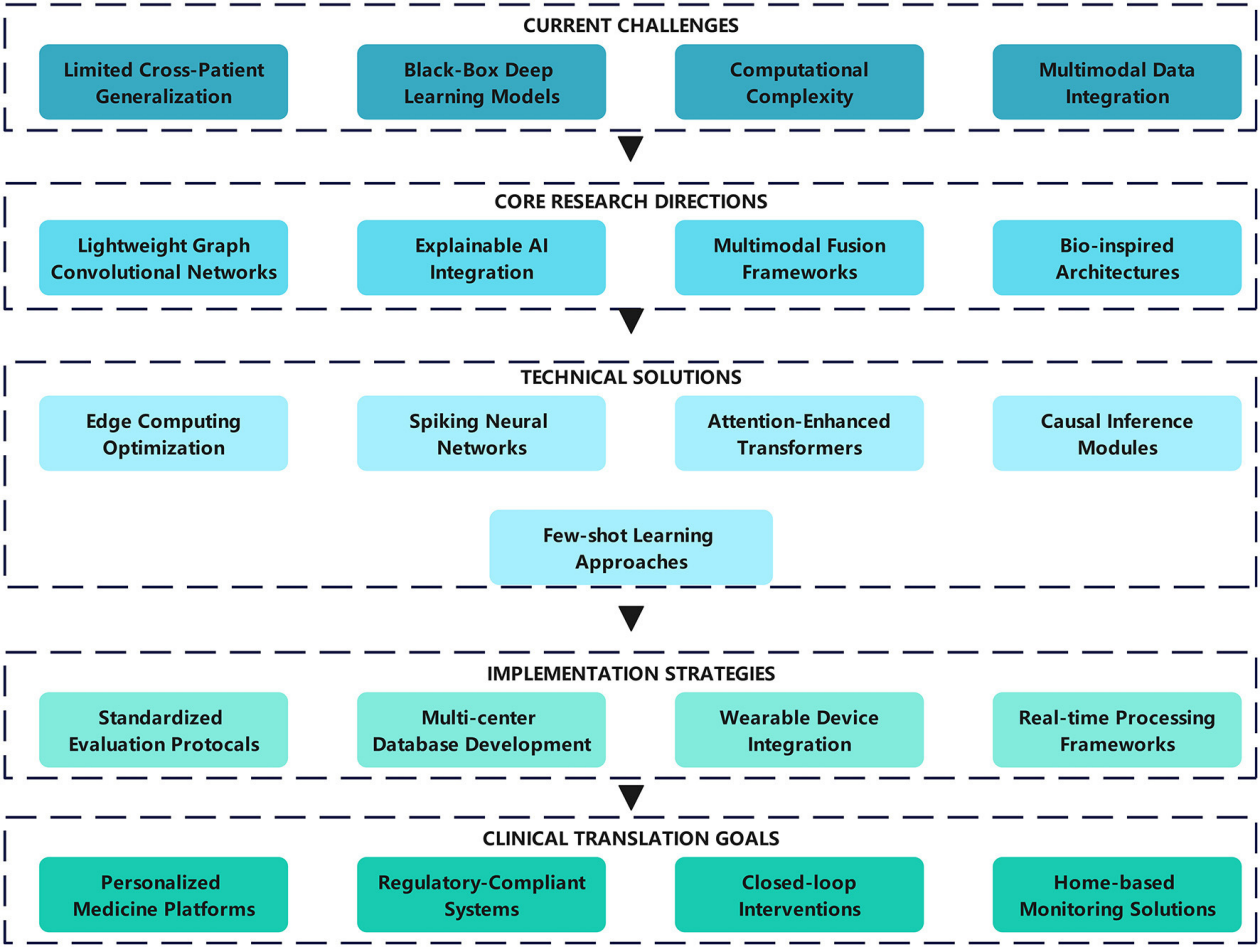
Future research directions in nonlinear dynamics-based epileptic EEG analysis.

Figure 6 clearly outlines the pathway toward personalized medicine, with the ultimate goal of developing clinically practical intelligent monitoring systems. This requires combining explainable AI technologies with embedded algorithms while adhering to medical compliance and ethical requirements. Through continuous technological innovation and interdisciplinary collaboration, epilepsy prediction technology will ultimately achieve a complete transition from theoretical research to clinical practice, providing patients with precise, personalized intervention solutions.

## 7 Conclusion

Nonlinear dynamics has fundamentally reshaped the research paradigm for epilepsy EEG analysis, providing critical insights to capture the complex dynamical shifts that precede seizures. Technical systems centered on hybrid models combining chaos theory metrics with deep learning (e.g., multiscale entropy-convolutional neural networks and attention-based long short-term memory networks) have achieved remarkable success in detecting pre-seizure nonlinear feature patterns. While these approaches demonstrate superior performance (achieving 15%–30% improvement over conventional methods), their clinical translation faces significant challenges due to dependence on expert-annotated data, insufficient interpretability, and performance variability in real-world applications. However, the field still faces significant long-term challenges including inconsistent evaluation criteria, insufficient validation in real-world scenarios, and limited handling of inter-patient variability. Although deep learning partially mitigates patient-specific variability through end-to-end feature learning, its dependence on highly annotated data and lack of model interpretability continue to constrain clinical deployment. To directly tackle the interpretability barrier, future developments must collaboratively build standardized multimodal databases, develop bio-inspired adaptive deep learning architectures (e.g., spiking neural networks and differential equation-driven dynamic networks), and construct regulatory-compliant evaluation frameworks. To address these validation gaps, emerging trends point toward edge computing wearable systems integrating multiscale entropy analysis with graph attention-augmented neural networks, promising closed-loop real-time interventions that can validate performance under actual ambulatory conditions. In particular, deep learning-driven predictive models need to be strengthened with causal inference modules to explain the associations between nonlinear features and pathological mechanisms, thereby meeting ethical scrutiny requirements. As technology advances, maintaining rigorous clinical outcome validation while addressing the ethical implications of predictive monitoring will be essential for driving the translation of these technological breakthroughs into substantial improvements in epilepsy diagnosis and treatment.

## Footnotes

^1^Available at: http://www.physinet.org/pn6/chnmit.

^2^Available at: http://epilepsy.uni-freiburg.de/freiburg-seizure-prediction-project/eeg-database.
